# Recent Advances in Flexible Ultrasonic Transducers: From Materials Optimization to Imaging Applications

**DOI:** 10.3390/mi14010126

**Published:** 2023-01-02

**Authors:** Danyang Ren, Yonggang Yin, Chiye Li, Ruimin Chen, Junhui Shi

**Affiliations:** Research Center for Humanoid Sensing, Zhejiang Laboratory, Hangzhou 311100, China

**Keywords:** flexible ultrasonic transducer, flexible single-element ultrasonic transducer, flexible array ultrasonic transducer, flexible micromachined ultrasonic transducer, imaging application, functional materials

## Abstract

Ultrasonic (US) transducers have been widely used in the field of ultrasonic and photoacoustic imaging system in recent years, to convert acoustic and electrical signals into each other. As the core part of imaging systems, US transducers have been extensively studied and achieved remarkable progress recently. Imaging systems employing conventional rigid US transducers impose certain constraints, such as not being able to conform to complex surfaces and comfortably come into contact with skin and the sample, and meet the applications of continuous monitoring and diagnosis. To overcome these drawbacks, significant effort has been made in transforming the rigid US transducers to become flexible and wearable. Flexible US transducers ensure self-alignment to complex surfaces and maximize the transferred US energy, resulting in high quality detection performance. The advancement in flexible US transducers has further extended the application range of imaging systems. This review is intended to summarize the most recent advances in flexible US transducers, including advanced functional materials optimization, representative US transducers designs and practical applications in imaging systems. Additionally, the potential challenges and future directions of the development of flexible US transducers are also discussed.

## 1. Introduction

An ultrasonic (US) transducer is a device that converts ultrasonic waves and electric energy into each other, and is often used in the field of ultrasonic imaging and photoacoustic imaging systems. US imaging systems are frequently used for industrial nondestructive testing and human health monitoring due to their subsurface inspection capabilities, simplicity, fast inspection speed, and ease of operation [1,2,3,4]. US transducers are used as both US emitting devices and detection devices in US imaging systems. A photoacoustic imaging (PAI) system, as a typical non-invasive and non-radiative biomedical imaging modality system, has the advantages of with high optical contrast images at a microscale resolution and a reasonable penetration depth due to the combination of optical excitation and US detection [5,6,7,8]. In the PAI system, the target tissues absorb optical energy after being irradiated by a pulsed laser and partially convert it into heat energy, resulting in a localized and rapid temperature rise and a subsequent generation of US waves. The generated US waves, also called PA signals, are usually detected by US transducers to form high-resolution PA images containing characteristic information of the tissues [9,10,11,12]. The image quality of the imaging system is influenced by several factors, such as spatial resolution and signal-to-noise ratio, which is determined by the performance of the US transducers.

Nevertheless, conventional US transducers for imaging applications are usually presented in rigid format, which may lead to limitations in some application scenarios, such as close contact with curved, complex and dynamic surfaces of the body and industrial sample [6,13,14]. The mismatch between the transducers’ rigid surfaces and irregular surfaces of the target tissues would generate an irregular, thick coupling layer between the above two surfaces, which leads to distortions and sensitivity loss. Compared to rigid US transducers, flexible US transducers provide the advantages of being thin, compact and lightweight and having the ability to conform to any complex surface [15,16,17]. Due to the superiority of the self-alignment to the target surface, even with curved or complex geometries, the flexible US transducer could detect the maximized transmitted US energy. Therefore, the flexible US transducer can be used to comfortably attach onto the target tissues to obtain continuous real-time and high-quality images in imaging applications. Additionally, the development of flexible US transducers is crucial to expand the application field of imaging. Recently, flexible US transducers with high performances in converting mechanical energy into electrical energy have attracted tremendous attention. Several groups have proposed some typical design strategies, such as attaching rigid piezoelectric material films to flexible substrates to construct flexible US transducers, directly fabricating flexible US transducers based on piezopolymers and flexible piezocomposites, embedding many rigid micromachined US transducer elements into flexible polymer substrates, and so on [16,17,18,19,20,21,22,23]. The recent boom in the development of flexible electronic technology has also laid the foundation for the fabrication of higher performance flexible US transducers, especially for flexible array US transducers.

In this review, the recent advances in flexible US transducers are summarized and classified, focusing on several representative flexible US transducers, as well as their corresponding functional materials optimization and device structure design. Furthermore, we also review the latest and representative flexible US transducers applied in the field of imaging applications, followed by a discussion of existing challenges and future directions in the development of flexible US transducers.

## 2. Advances in Functional Materials for Flexible US Transducers

Flexible US transducers consist of several vital component functional materials, such as high-performance piezoelectric materials, which can convert mechanical energy and electrical energy into each other, flexible substrate materials, which can adapt to any shape, and deformable electrical conductor materials, which can maintain a good conductive effect in the deformed state. In flexible US transducer design, several material properties should be considered, including piezoelectric properties, electromechanical coupling properties, acoustic impedance, electrical conductivity and flexibility. In this section, we focus on the design, fabrication and application of functional materials for flexible US transducers.

### 2.1. High Performance Piezoelectric Materials

Piezoelectric material, which is used as an active element to convert mechanical energy to electrical energy, is the core component of the flexible US transducer. Therefore, the selection of the piezoelectric materials for flexible US transducers is mainly based on the piezoelectric constants, electromechanical coupling coefficient, acoustic impedance and flexibility of the materials. A high electromechanical coupling coefficient (k) and a low acoustic impedance ($Z_{a})$ are both favorable parameters of piezoelectric materials for flexible US transducers [23]. When we designed single-element or array transducers, several kinds of piezoelectric materials with their unique performances could be chosen depending on specific requirements, including flexibility, bandwidth and sensitivity [21,24]. At present, there are three main methods to fabricate the core flexible active materials for flexible US transducers. First, fabricating flexible piezoelectric polymers directly to be used as active materials for flexible US transducers [25,26]. Second, attaching rigid piezoelectric material films on flexible substrates to form the active materials with certain flexibility [1,19,27]. Third, assembling many tiny units of rigid piezoelectric materials on/into a flexible polymer substrate to prepare the flexible piezocomposites as active materials [21,24,28]. However, all methods have their own disadvantages and advantages, so we should weigh them when deciding which one to choose.

**Figure 1:**
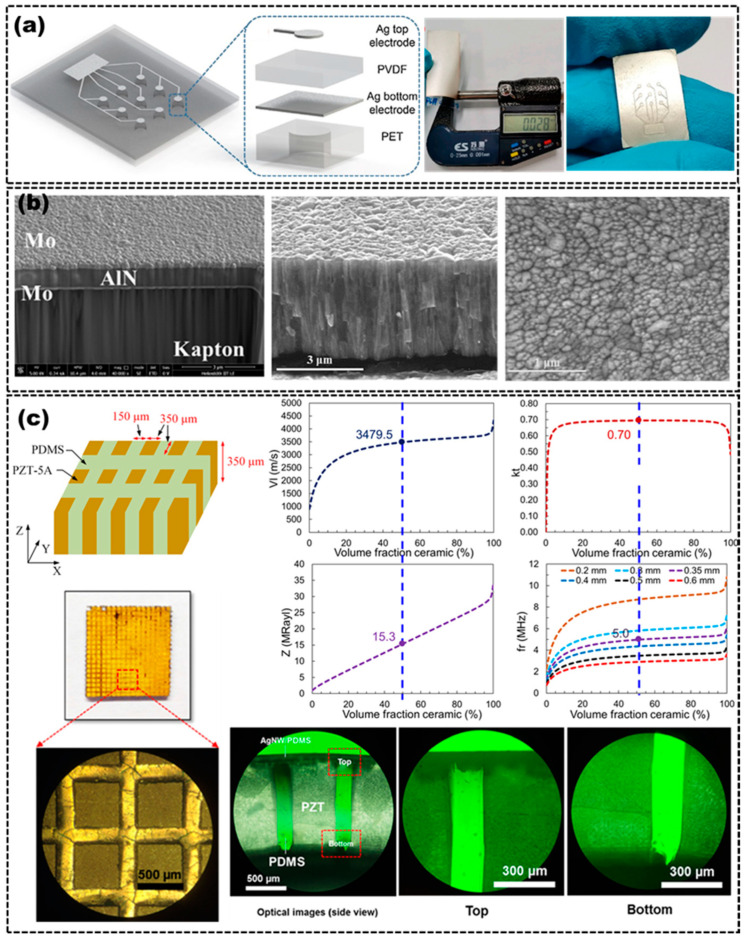
(**a**) Schematic of the cross-section of the transducer based on PVDF film (**left**), photograph of the PVDF (**middle**) and transducer (**right**). Adapted from [29,30]. (**b**) Scanning electron microscopy (SEM) images of the cross-section of the AlN film deposited on the substrate (**left**), cross-section of the ZnO film (**middle**) and surface of the ZnO film (**right**). Adapted from [19,27]. (**c**) Schematic diagram of the 1-3 piezocomposite (**top left**), curve of effective material properties of 1-3 piezocomposites with the volume fraction of PZT (**top right**), optical images of the 1-3 piezocomposite after deposition of the Au electrode (**left bottom**) and the interface between the PZT and PDMS (**bottom right**). Adapted from [21,24].

In order to ensure the long lifetime of flexible US transducers, it is a good approach to directly use piezoelectric polymers as active materials to fabricate the transducers [29,30,31,32]. Compared with rigid piezoelectric materials such as piezoelectric single crystal, piezoceramics and some rigid piezocomposites, piezoelectric polymers have a significantly lower acoustic impedance (~4 MRayl) match to body tissue (1.5 MRayls) [32], broad bandwidth and inherent flexibility, which are all key factors for fabricating flexible US transducers for PAI applications. Liu et al. fabricated a flexible US transducer by employing a PVDF thin film as the active piezoelectric layer and indium tin oxide as the electrodes, which could fit perfectly on concave, convex, and complex surfaces, as shown in Figure 1a [29,30]. The flexible US transducer showed a center frequency of 6.7 MHz and a fractional bandwidth of 86.3% (−3 dB). However, the low electromechanical coupling coefficient of the piezoelectric polymer limits the sensitivity of the flexible US transducer to some extent.

Compared with piezoelectric polymers, rigid piezoelectric materials, such as piezoceramics and piezoelectric single crystals, have excellent electromechanical coupling properties to ensure the sensitivity of the transducers. Although conventional bulk rigid piezoelectric materials could not be used as the active materials for flexible US transducers due to their inherent brittle and high stiffness, depositing thin films of rigid piezoelectric materials on flexible substrates can reduce the inherent brittleness of these bulk materials and render them a degree of flexibility, which could be used as active materials for flexible US transducers [16]. There has been an increased interest recently in the development of flexible US transducers using high performance piezoelectric thin film materials, such as AlN, ZnO and PZT, as shown in Figure 1b [19,27]. These materials could be deposited on flexible substrates, such as thin metallic foils, to prepare flexible US transducers [16,18,19,27,33], due to the inherent physical flexibility of the film or the ultrathin thickness weakening its inherent stiffness. Vincenzo et al. reported a flexible US transducer, which was based on micromembranes of piezoelectric AlN embedded between two Molybdenum electrodes on a Kapton substrate [27]. Hou et al. designed a flexible US transducer consisting of a 3 μm thick piezoelectric ZnO film layer on a 50 μm thick Al foil substrate, which had a center frequency in range of 24–29 MHz, with a bandwidth of 4–7 MHz at –6 dB [33]. However, the sputter-deposed AlN or ZnO films of a few microns usually have a large film stress and many defects, which may have a significant impact on the performances of the flexible active film, thereby limiting the application of the US transducers. Kobayashi et al. used the sol–gel spray technique to fabricate 40 μm thick PZT/PZT films on stainless steel, which serve as both the substrate and the bottom electrode [1]. The flexibility was achieved due to the porosity in the PZT/PZT films and the thinness of the PZT/PZT films and stainless steel. The flexible US transducer made of PZT/PZT film had a 13.1 MHz center frequency and a 10.2 MHz bandwidth at –6dB, which displayed signal strengths and the bandwidth is both comparable to commercial transducers. However, the electromechanical coupling coefficient ($k_{t}$) of the thick PZT/PZT film was 0.24 times lower than the value of the bulk PZT ($k_{t}\sim 0.5)$, which would affect the conversion efficiency of electrical energy and acoustic energy of the flexible active material. Moreover, active materials composed entirely of piezoceramics have a high acoustic impedance, which affects the transmission efficiency of the US signal and even the sensitivity of the US transducer.

To enhance the electromechanical coupling property and reduce the acoustic impedance of the flexible active material, flexible piezocomposites have emerged because of their excellent tailorable properties between rigid piezoelectric materials and flexible polymers. The piezocomposites usually consist of two phases of rigid piezoelectric materials and flexible polymers to tune their properties, displaying superior properties when compared to single-phase materials. Piezocomposites, made up of two phases, have a variety of connectivity modes, which can be divided into 0–3, 1–3, 2–2 and other structures according to the connectivity of each phase (continuous in zero, one, two, or three dimensions) [34]. The connectivity modes within the piezocomposites are governed by the arrangement of the phases composing the piezocomposites, which would determine the electromechanical coupling properties of the piezocomposites. 1-3 piezocomposites, formed by embedding rigid piezoelectric rods in a polymer matrix, are more widely used in the fabrication of flexible US transducers [21,24]. In some cases, the thickness-mode electromechanical coupling coefficient of the piezocomposites can exceed the $k_{t}$ of rigid piezoelectric materials, almost approaching the value of the rod mode, $k_{33}$ of rigid piezoelectric materials. Additionally, the acoustic impedance can be reduced by replacing the dense and stiff rigid piezoelectric materials with a low density and soft polymer. Gerald et al. used an extensive finite element modeling to design a 1-3 piezocomposite structure as the flexible active material for US transducers, which was fabricated by dispersing piezoceramic (PZT–5A) fibers randomly into a polymer (epoxy) matrix [35]. Taeyang et al. proposed a 1.8 MHz flexible US transducer using a PZT–5H/polydimethylsiloxane (PDMS) 1-3 piezocomposite as the flexible active material. The 1-3 piezocomposite was made by dicing bulk PZT to form the pillars and filling the resulting kerfs with PDMS as an interstitial material [21]. The arrangement provided sufficient separation of the miniature rigid piezoceramic units in the flexible substrate, which ensures the flexibility of the active material and maintains the high electromechanical coupling coefficient of the piezoceramic and the low acoustic impedance of the polymer. The electromechanical coupling coefficient and the acoustic impedance are 0.74 and 19.2 MRayl, respectively. Additionally, the degree of the flexibility and the performances of the 1-3 piezocomposites were determined by the volume fraction of the rigid piezoelectric materials and the material properties of the rigid piezoelectric materials and polymers, as shown in Figure 1c [21,24]. US transducer designers can tailor the piezocomposites’ performances according to the requirement of specific applications by varying the materials proportion. Unfortunately, it is almost impossible to optimize all parameters at the same time, so a compromise solution is often chosen when designing composites. For instance, Peng et al. also reported a PZT/PDMS 1-3 piezocomposite with a different volume fraction from that of Taeyang’s [24], which caused the properties of the piezocomposite and US transducer to exhibit a few differences. The electromechanical coupling coefficient and the acoustic impedance of the 1-3 piezocomposite are 0.68 and 13.37 MRayl, respectively. After adjusting the ratio of the rigid material to the polymer, the acoustic impedance of the piezocomposite is lower than that of Taeyang’s, but its electromechanical coupling coefficient is also sacrificed.

Briefly, using piezopolymers directly as flexible active materials allows the flexible US transducer to achieve a broad bandwidth and the longest lifetime, because of the inherent flexibility of the polymers. Moreover, the significantly low acoustic impedance closely matched that of body tissue for strong acoustic coupling and minimization of the reflection from the interface of the transducer and skin. However, the low electromechanical coupling coefficient would limit the conversion efficiency of electrical and acoustic energy, and, thus, lower the sensitivity of the flexible US transducers. The flexible active materials formed by depositing rigid piezoelectric material films usually have a low electromechanical coupling coefficient and high acoustic impedance, which are not suitable for the fabrication of US transducers other than high-frequency transducers. The piezocomposites, produced by assembling many tiny rigid piezoelectric material units into a flexible polymer substrate, can maintain a high piezoelectric constant, high electromechanical coupling coefficient and low acoustic impedance. However, the rigid material may separate from or even fall off the flexible substrate after undergoing thousands of deformations.

### 2.2. Flexible Substrate Material for Flexible US Transducers

**Figure 2:**
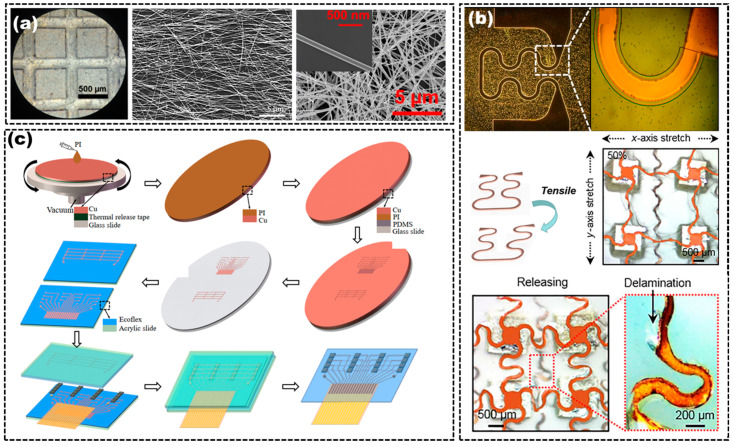
(**a**) Optical image of the AgNW electrode on the transducer (**left**), SEM image of the AgNW film (**middle**) and SEM image of AgNWs at high magnifications (**right**). Adapted from [21,24]. (**b**) Electrode patterned as serpentine structures (**top**), schematic diagram of electrode stretching (**middle left**), optical images of the electrode in stretched (**middle right**) and relaxed states (**bottom**). Adapted from [23,42]. (**c**) Fabrication process of patterning stretchable electrodes with a laser. Adapted from [44].

**Figure 3:**
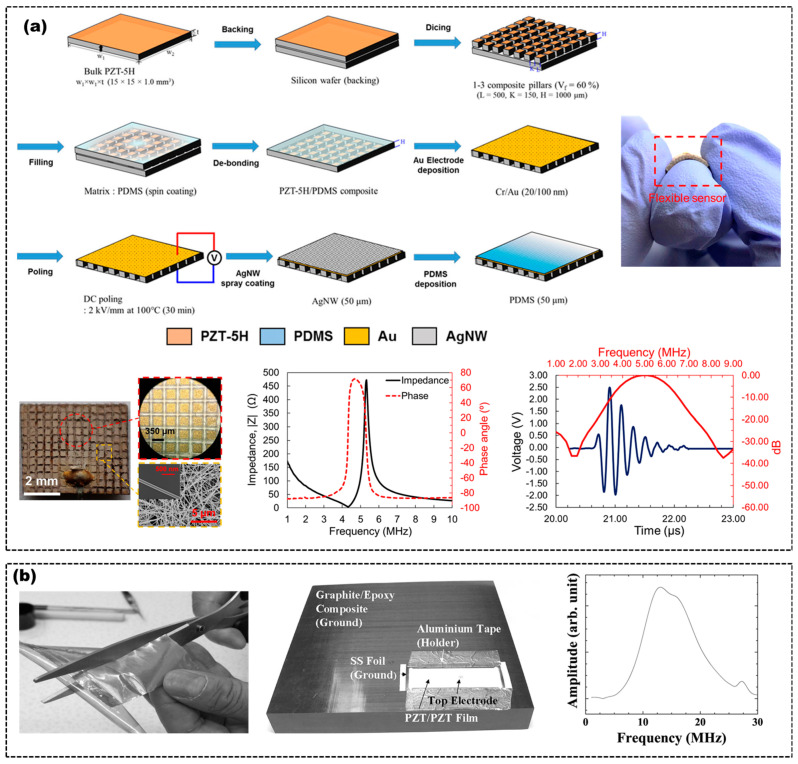
(**a**) Fabrication process of a flexible single-element transducer based on PZT/PDMS 1-3 piezocomposite (**top left**), photographs (**bottom left**), flexibility test (**top right**), electrical impedance and phase angle (**bottom middle**) and response waveform and frequency spectrum (**bottom right**) of the transducer. Adapted from [21,24]. (**b**) Photograph of a ZnO/Al foil flexible active material (**left**), photograph (**middle**) and frequency spectrum (**right**) of a PZT/PZT flexible transducer. Adapted from [1,33].

**Figure 4:**
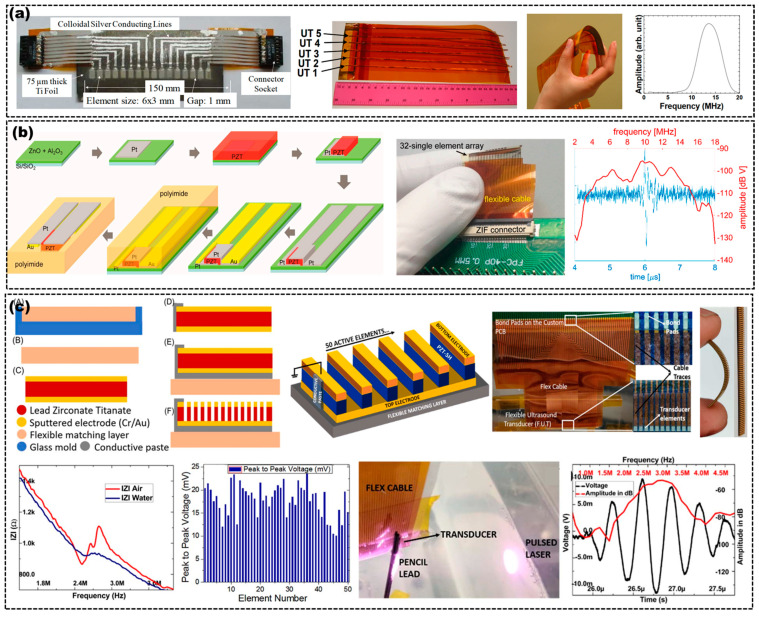
(**a**) Top view of a 1−D linear flexible US transducer based on PZT (**left**), optical images (**left second**), flexibility test (**left third**) and frequency spectrum (**right**) of a flexible PZT/PZT 5−element 1−D array. Adapted from [36,38]. (**b**) Fabrication process of a single element (**left**), optical image (**middle**) and performance (**right**) of a 32−element 1−D array based on PZT. Adapted from [16]. (**c**) Fabrication process (**top left**), 3D schematic (**top second**), optical images (**top right**) and performances (**bottom left, second** and **right**) of a flexible 1−D array, a flexible 1−D array with flexible PCB (**top third**) and experimental setup used for the obtained PA signals from pencil lead (**bottom third**). Adapted from [55].

**Figure 5:**
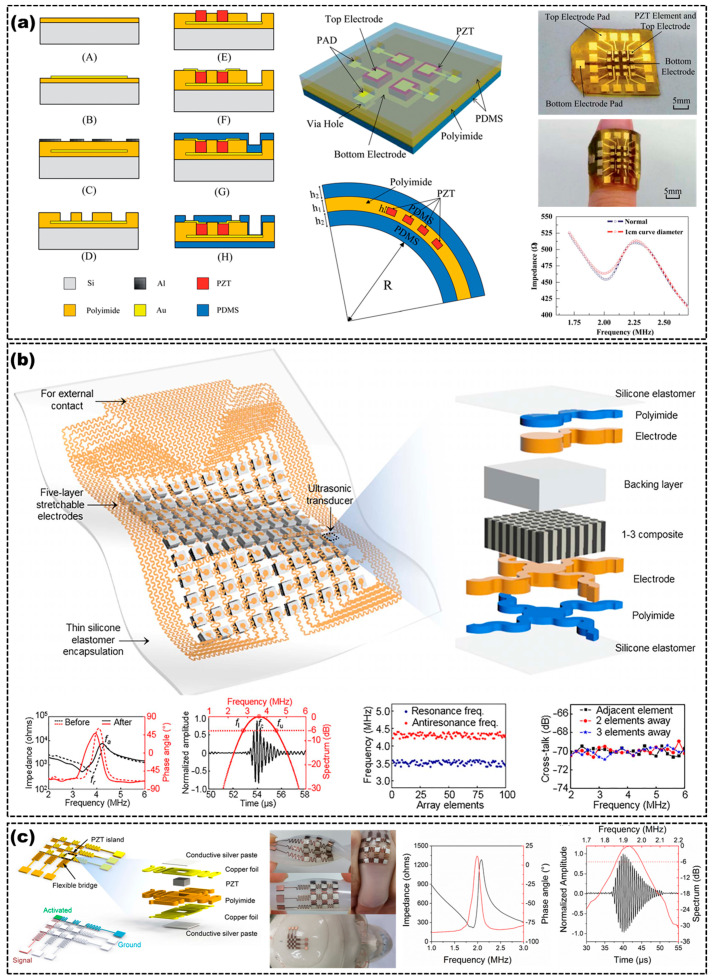
(**a**) Fabrication process (**top left**), illustration (**middle**), photographs (**top right**) and impedance curve in different states (**bottom right**) of the 2− flexible array transducer. Adapted from [39]. (**b**) Schematics (**top**), impedance curve (**bottom first**), frequency spectrum (**bottom second**), resonance and antiresonance frequency variations (**bottom third**) and average cross-talk levels (**bottom fourth**) of a 2−D flexible array US transducer. Adapted from [23]. (**c**) Schematics (**left**), optical images (**second left**), impedance curve (**third left**) and frequency spectrum (**right**) of the flexible 2−D array US transducer. Adapted from [17].

The flexible substrate, a key factor determining the flexibility of the transducer, is typically used as the deformable platform to be embedded/integrated by piezoelectric materials, electrical conductors or transducer arrays. The flexible substrate is usually engineered from deformable and flexible polymer materials, or ultrathin metallic films, such as elastomers, soft polymer films and stainless-steel films, as shown in Figure 2, Figure 3 and Figure 4 [36,37,38,39]. Elastomers have been used as flexible and stretchable substrates for flexible US transducers due to their natural flexibility and elasticity. Among elastomers, PDMS is highly flexible and capable of sustaining stretchability greater than 170% in tensile strain [21,24,39,40], which allows it to not only act as the flexible substrate embedded by rigid piezoelectric material rods but also as the packaging material to encapsulate transducer devices. Taeyang and co-workers used PDMS as the polymer matrix to integrate rigid piezoceramic rods to achieve excellent flexibility of the active material for flexible US transducers, which could obtain excellent flexibility, a broad bandwidth and sensitivity [21], as shown in Figure 3a. Wang et al. proposed flexible 2-D piezoelectric micromachined US transducer arrays at a center frequency of 2 MHz, which was made on a polyimide (PI) substrate and packaged with PDMS [20], as shown in Figure 5a. PI is a typical soft polymer, which has good biocompatibility, a high tensile strength and high flexibility [36,41,42]. Additionally, the natural, excellent mechanical properties of the soft polymer make it easy to function well under a bending deformation state when used as the flexible substrate for flexible US transducers. In addition, other deformable and flexible polymer materials, such as epoxy, polyethylene terephthalate (PET), polyurethane (PU) and silicones, have also demonstrated outstanding flexibility to be able to act as the flexible substrates for flexible US transducers [41,42,43].

### 2.3. Flexible Electrical Conductors and Technologies

The development of flexible US transducers, combining flexible electronics and US technology, is also closely related to the advances in flexible and miniaturized electronics. Conventional metal-type and metal foil electrical conductors limit the development of wearable and stretchable electronics due to a non-flexible nature, cracks or delamination after deformation and weak bonding forces with the active materials. With the increasing demand for wearable and stretchable electronics, such as wearable sensors, stretchable supercapacitors and flexible US transducers, research on flexible electrical conductors and technologies has gradually emerged in recent years [22,45,46]. The development of electrical conductors and technologies also facilitate the fabrication of flexible US transducers and broaden their applications, due to their abilities to maintain electrical conductivity after undergoing mechanical deformation. For single flexible US transducers, the flexible electrical conductors act as the electrodes of the active materials. For flexible US arrays, flexible electrical conductors have been used as both the electrodes and the interconnects to fabricate the electrical connection of the matrix of active material elements or US transducer elements in flexible arrays. Flexible electrical conductors have been designed and fabricated by several methods, such as preparing functional electrical conductor materials directly and designing electrical conductor geometric structures.

There are two common methods for designing functional electrical conductor materials, including directly using conductive polymers and using conductive composites formed by filling conductive phase into the elastomer matrix. Poly(3,4-ethylenedioxythiophene) polystyrene sulfonate (PEDOT:PSS), polyaniline (PANI) and polypyrrole (PPy) are typical conductive polymers which have been widely used as flexible electrodes for sensors and supercapacitors due to their high flexibility and electrical conductivity [45,46]. However, few researchers have directly used conductive polymers as electrodes of flexible US transducers due to their low conductivity (<700 S/cm) and electromechanical effect (i.e., electrically induced volume change), which are limitations as electrodes for US transducers [45]. Conductive composites, composed of the elastomer matrix and conductive fillers, have already been used as the electrodes in flexible US transducers because of their high flexibility, high conductivity and simple processability. PDMS is a type of elastomer and shows a dielectric constant in the range of 2.8–3.2, which also allows it to be used to improve the elasticity and flexibility of some electrical conductors. Taeyang et al. deposited a mixture of sliver nanowires (AgNWs) and PDMS as the flexible electrodes (AgNW/PDMS), which was formed by embedding the AgNWs into the PDMS substrate. The AgNW/PDMS electrodes showed excellent conductivity (>5000 S/cm) and low resistance (<5 Ω). Compared with the traditional Au electrodes, the AgNW/PDMS electrodes could provide sufficient electrical conductivity after undergoing continuous deformation and they had reliable durability to the cracks from the strained fatigue, as shown in Figure 2a [21,24].

Designing electrical conductor geometric structures based on conventional rigid electrode materials is another common strategy to render flexibility to the electrical conductors. Ultrathin ribbons and filaments in the forms of serpentine shapes are typical geometric structures used to fabricate flexible electrical conductors with rigid conductive materials [47]. The designed geometric structures could be fabricated by machining, printing and micro-nanofabrication [48,49]. Metals, as the representative rigid conductor materials, have an inherent excellent conductivity, and are usually used to design the geometric structures to be used as flexible electrical conductors. Liu et al. used Cu to design double-sided conductive serpentine-shaped wires as the flexible electrical conductors. They regarded the piezoelectric unit as an “island” and the electrical conductor as a “bridge” to connect the islands. They used the serpentine joints to connect piezoelectric islands to each other. The addressable electrode arrangement is designed to effectively excite and connect nine units and comply with the single-layered “island-bridge” layout [17], as shown in Figure 5c. Xu et al. fabricated a multilayer material composed of 20 µm thick Cu foil and 10 µm thick PI film, used the laser to pattern serpentine-shaped electrical conductors, peeled off the excess Cu-PI layer and finally used it as the flexible electrical conductors for flexible US transducers, as shown in Figure 2c [44]. Liu et al. used photolithography and etching to make Cu foil into a serpentine-shaped structure to be used as the flexible electrical conductors, as shown in Figure 2b [42]. However, the performance of the flexible electrical conductors prepared by the designed structure is limited by several factors, such as the thickness of the ribbons or filaments, the adhesion of the ribbons or filaments with substrates and the flexibility of the substrates. Moreover, complex flexible electrical conductor structures and manufacturing technologies enhance the instability and the risk of the fabrication process of the flexible US transducers.

## 3. Flexible US Transducer Designs for Imaging Applications

### 3.1. Flexible Piezoelectric Transducers

Recent advances in wearable devices provide innovative materials and manufacture process making it possible to produce flexible US transducers for imaging applications. Like rigid US transducers, common types of flexible US transducers also include single-element and array US transducers. Compared to flexible US array transducers, the flexible single-element US transducers have a low complexity, a simple fabrication process and a high center frequency. Fabricating the flexible single-element US transducers based on the piezoelectric film (i.e., ZnO, AlN, PZT and PVDF) and the flexible piezocomposites is the most promising strategy [50,51]. Kobayashi et al. and Hou et al. designed a flexible single-element US transducer based on PZT or ZnO thin film, with a center frequency of 13.1 MHz and 29 MHz, respectively [1,33], as shown in Figure 3b [1,33]. However, these flexible single-element US transducers were prepared with no acoustic matching layers and backing block, which will affect the sensitivity and narrow the bandwidth. Peng et al. reported a flexible single-element US transducer by the “dice and fill” technology, which used the PZT−5H/PDMS 1-3 piezocomposite as the flexible active material and the Ag/PDMS composite layer as the flexible electrical conductors [24]. The 1-3 piezocomposites usually have a lower acoustic impedance than that of PZT film, so that the wide bandwidth transducer could be fabricated without an acoustic matching layer. The fabrication of this transducer is similar to the process shown in Figure 3a [21]. The flexible US transducer showed excellent flexibility, a broad bandwidth (~47%) and a center frequency of 5 MHz, as shown in Figure 3a.

However, flexible single-element US transducers must be mechanically scanned to form the images, which is disadvantageous for real-time imaging in imaging systems. The flexible array US transducers with multichannel electronics can obtain images by fast electronic scanning without mechanical scanning. Additionally, the imaging systems with flexible array US transducers can provide better real-time imaging ability compared to that of flexible single-element US transducers. The development of flexible array US transducers enables the generation of real-time volumetric imaging, which could provide a better visual representation of target tissues with complex geometrics. The frequency and bandwidth of the flexible array US transducers determine the axial resolution of the imaging applications, and the aperture width and element directivity of the array transducers have a significant impact on the lateral resolution. Moreover, the array interelement spacing and the total number of array elements can also impact the final image quality of the imaging systems. There are several types of common flexible array US transducers, such as the one-dimensional (1−D) linear array, the two-dimensional (2−D) matrix array and the capacitive micromachined US transducer (CMUT) array.

The 1−D linear flexible array US transducers are generally composed of multiple rigid US transducer elements connected in sequence through polymers or cables [28,41,52,53,54]. Several methods have been attempted to fabricate the 1-D flexible array US transducers. Casula et al. reported 1−D flexible array US transducers composed of 24 rigid US transducer units of 1.3 × 20 mm^2^, mechanically assembled with cables to obtain a jointed structure [52]. The pitch and area dimensions are 2 mm and 48 × 20 mm^2^, and the center frequency is 2 MHz. The 1−D array could deform its shape up to concave or convex surfaces according to the shape of the target tissue. Bowen et al. fabricated a 1−D flexible array US transducer by placing the 800 μm diameter PZT−5A piezoelectric fibers in parallel with an 800 μm air gap separating them, and the air gap ensured the flexibility normal to the fiber direction [28]. Shih et al. prepared a 16−element 1−D linear flexible array transducer, which was made by a technology of a PZT sol–gel sprayed piezoelectric thick film on Ti foil, as shown in Figure 4a [38]. Each element size of the 1−D array is 6 × 3 mm^2^ and the gap between two adjacent elements is 1 mm on 75 μm thick Ti foil. Additionally, the center frequency of each element was about 6 to 7 MHz. Kobayashi et al. fabricated a 1−D linear flexible array US transducer, which consisted of a thin PI membrane with a bottom electrode or stainless-steel foil, PZT composite film and top electrodes, as shown in Figure 4a [36]. Liu et al. reported 9.5 MHz 1−D linear flexible PZT-based US transducer units on the PI substrates, which consisted of 32 transducer units [16]. The photograph picture and the performance of the 1−D array are displayed in Figure 4b. Roy et al. fabricated a 50−element 1−D linear flexible US array by using PZT as the active material, epoxy as the matching layer and flexible substrate, conductive epoxy as the binder for PZT and flexible substrate [55]. Schematics, photographs and performances of the flexible US transducer are shown in Figure 4c [55]. The 1−D linear flexible array US transducers are often used for inspection of target tissues with concave, convex or tubular shapes. Although the imaging systems using the 1−D linear array transducer can achieve real-time imaging, the electronic sequencing or scanning of it must be repeated fast enough.

Compared to a 1−D flexible array, 2−D matrix flexible array US transducers combine the advantages of conformability and real-time imaging better, which could be used to inspect the target tissues with irregular geometrics and more complex surfaces. By using 2D arrays, cylindrical volumes can be scanned, providing vertical and horizontal views of the test objects without the need to switch transducers or operations, and reducing the scanning time. Accordingly, the design and manufacturing process of 2−D arrays are more complex than those of 1−D arrays [56]. Yang et al. fabricated a 4 × 4 array flexible transducer with a unit size of 1mm. The fabrication process, illustration, photographs and impedance results of the 2−D flexible array transducer are shown in Figure 5a [39]. Hu et al. produced a 10 × 10 array unit 2−D matrix flexible array US transducer that could conform to and detect complex surfaces [23]. The transducer used several thin and high-performance PZT/epoxy 1-3 piezocomposites as the transducer units, 20 μm thick multilayered serpentine Cu traces as electrical conductors, and PDMS elastomer membranes as encapsulation materials. The schematics and design of the 2−D array flexible US transducer are displayed in Figure 5b [23]. Although the transducer units were rigid, the serpentine electrical interconnects ensured the overall flexibility of the 2−D arrays. The flexible electrical interconnects were covered by a 2 μm thick PI layer to enhance the bonding strength between Cu and PDMS. Additionally, they used a Ag-epoxy composite as the backing layer to dampen the ringing effects of the piezocomposites, which could shorten the spatial pulse length and broaden the bandwidth and, thereby, improve the image axial resolution. The 2−D array required no matching layer due to the low acoustic impedance of the 1-3 piezocomposites. The 2−D matrix flexible array US transducer had a high electromechanical coupling coefficient ($k_{eff}\sim 0.6$), a high signal-to-noise ratio (SNR~20.28 dB), a wide bandwidth (~47.11%) and a negligible cross-talk level between adjacent elements (~−70 dB), as shown in Figure 5b. Additionally, the combination of performances ensured the high sensitivity of the imaging system. Liu et al. also fabricated a similar 3 × 3 array 2−D flexible US transducer, which used PZT as the active material of the transducer units, as shown in Figure 5c [17].

### 3.2. Flexible Micromachined US Transducers

**Figure 6:**
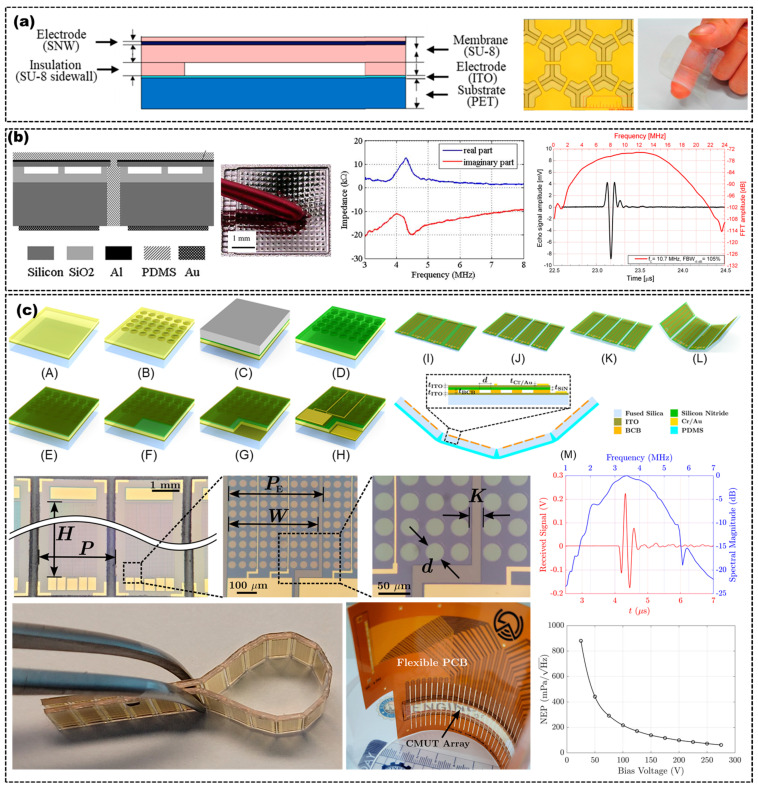
(**a**) Cross-sectional view of a CMUT unit (**left**), optical images (**right**) of a flexible CMUT array. Adapted from [57]. (**b**) Cross-section schematic of two flexible CMUT array elements (**first**), optical picture (**second**) and electrical impedance spectrum (**third**) of a 2−D CMUT array and frequency spectrum of a single element of a flexible CMUT array (**fourth**). Adapted from [58,59]. (**c**) Fabrication process and 2D cross-sectional view (**top**), optical images (**middle** and **bottom left**), frequency spectrum (**middle right**) and NEP (**bottom right**) of a transparent flexible CMUT array. Adapted from [15].

With the advances in micro-electro-mechanical-system (MEMS) technology, the capacitive micromachined US transducer (CMUT) array has emerged in recent years. The CMUT consists of a plurality of electrostatically driven micromembranes connected in parallel, which are fabricated on a substrate using surface micromachining technology. Additionally, each electrostatic cell has a vibrating membrane with a top electrode, and it is separated from the fixed bottom electrode by a vacuum cavity. The typical structure of the CMUT in cross-sectional view is shown in Figure 6a. The membrane can be driven by the electrostatic force produced by a voltage signal applied between the electrodes, which generates the US waves. On the contrary, when US waves vibrate the membrane, this will cause a change in the capacitance. At this time, the US waves can be detected by detecting the voltage or the current of the CMUT. The CMUT uses integrated circuit fabrication techniques to achieve electromechanical coupling coefficients almost as high as piezoelectric materials. Compared with typical piezoelectric transducers, CMUT offers a better matching to load, provides a larger bandwidth and exhibits a higher sensitivity, making its use attractive in imaging systems. Integrating several miniaturized CMUT units on a flexible substrate or fabricating the entire CMUT arrays based on flexible materials are also the two typical methods for designing flexible CMUT arrays.

Pang et al. presented a transparent flexible CMUT array, which comprises a tin oxide-PET (ITO-PET) substrate, an SU−8 sidewall and vibrating membranes, and a sliver nanowire electrode, as shown in Figure 6a [57]. Chen et al. reported flexible CMUT arrays, which exhibited a 4.5 MHz center frequency with a –3 dB fractional bandwidth of 82% [60]. Zhuang et al. proposed a technique for constructing flexible CMUT arrays by etching through-wafer trenches to isolate array elements and refilling the trenches with PDMS [58,61]. The 4.4 MHz resonant frequency flexible CMUT arrays consisted of a 6~20 μm-width trench, a 150 μm depth trench, a 1.83 μm thick membrane and a 150 μm thick substrate. The partial cross-section schematic, photos and electrical impedance spectrum of the flexible CMUT arrays elements is shown in Figure 6b [58]. Caronti et al. fabricated a flexible CMUT arrays by embedding CMUT die with a total thickness of only 6.5 μm into flexible backing and coating layers [59]. The flexible CMUT arrays exhibited a center frequency of 11 MHz with a fraction bandwidth of at least 100%, with no apparent degradation of properties resulting from the deformation of the array, as shown in Figure 6b [59]. Mahyar et al. proposed a flexible transparent CMUT array with a center frequency of 3.5 MHz, an 80% fractional bandwidth and a noise equivalent pressure (NEP) of 62 $\mathrm{mPa}/\sqrt{\mathrm{Hz}}$. The flexible CMUT array was fabricated based on an adhesive bonding technique with benzocyclobutene (BCB) as both the adhesive and sidewall layers, ITO as the electrodes, silicon nitride as the membrane, and PDMS as the flexible backing layers [15]. The fabrication processes, optical microscopy images, photographs, NEP and frequency spectrum of the transparent flexible CMUT array are shown in Figure 6c.

**Figure 7:**
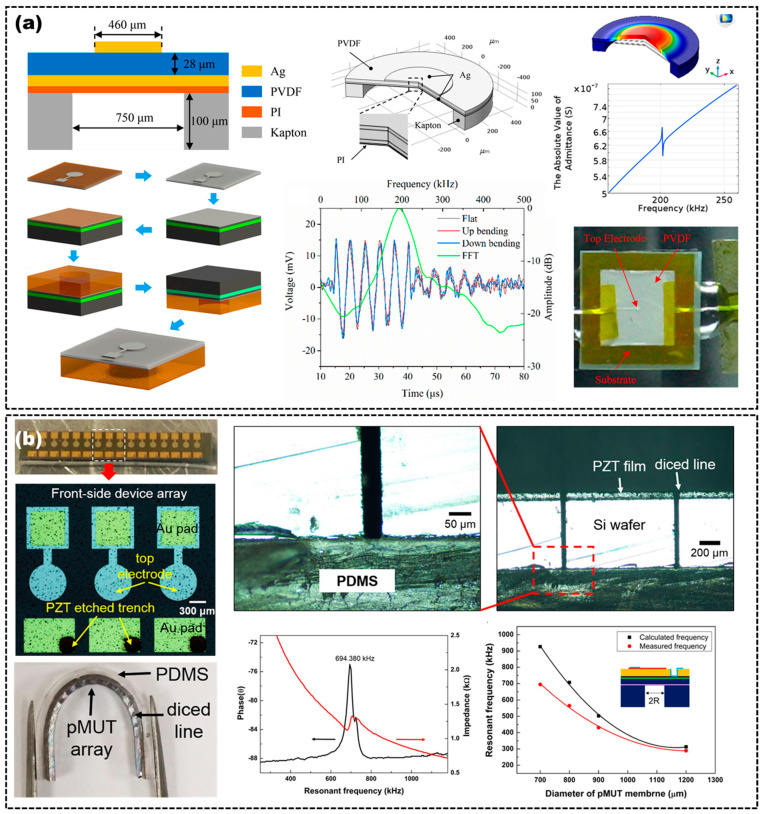
(**a**) Cross-sectional view of a PMUT unit (**top left**), finite element analysis of the PMUT (**top right**), schematic illustration of the fabrication process (**bottom left**), spectrum of the receiving signals (**middle bottom**) and optical image of the PMUT (**bottom right**). Adapted from [29]. (**b**) Optical image of a PMUT array (**left**), cross-sectional microscopy image of a PMUT array on PDMS after the dicing process (top right), phase and impedance results of a PMUT (**middle bottom**) and resonant frequencies of the PMUT with different membrane diameters (**bottom right**). Adapted from [62].

A flexible piezoelectric micromachined US transducer (PMUT) is also a typical application of MEMS technology, which consists of a top electrode, a piezoelectric layer, a bottom electrode, a structural layer and a flexible substrate [62]. The PMUT is driven by the piezoelectric layer and transmits and receives US waves via the bending diaphragm [2,42,62]. Therefore, PMUT can overcome the difficulties of manufacturing complexity and miniaturization of traditional piezoelectric ultrasonic transducers, such as the acoustic impedance mismatch between the piezoelectric layer and the propagation medium, the precise matching of the piezoelectric layer thickness and the large volume of the lining layer. Compared to CMUT, PMUT can obtain a higher sensitivity by improving its coupling coefficient [63,64]. However, the center frequency of existing flexible PMUT is generally lower than the megahertz level [29,30,42]. Liu et al. fabricated a flexible PMUT, which consists of an Ag-coated PVDF film mounted onto a laser-manipulated polymer substrate, as shown in Figure 7a [62]. They used the PI as the passive layer and bonded layer simultaneously, and used the Kapton film as the substrate to form a suspended structure of composite plates. The PMUT has a center resonant frequency of 198 kHz with a wide operational bandwidth. Lee et al. proposed a flexible PMUT array with an input resonant frequency of 694.4 kHz. They used PZT as the piezoelectric layer to make diaphragm US transducer arrays, then used oxygen plasma treatment to firmly bind the US transducer arrays to the PDMS substrate, and then precisely diced with a fixed pitch to achieve flexibility, as shown in Figure 7b [62].

## 4. Practical Imaging Applications with Flexible US Transducers

**Figure 8:**
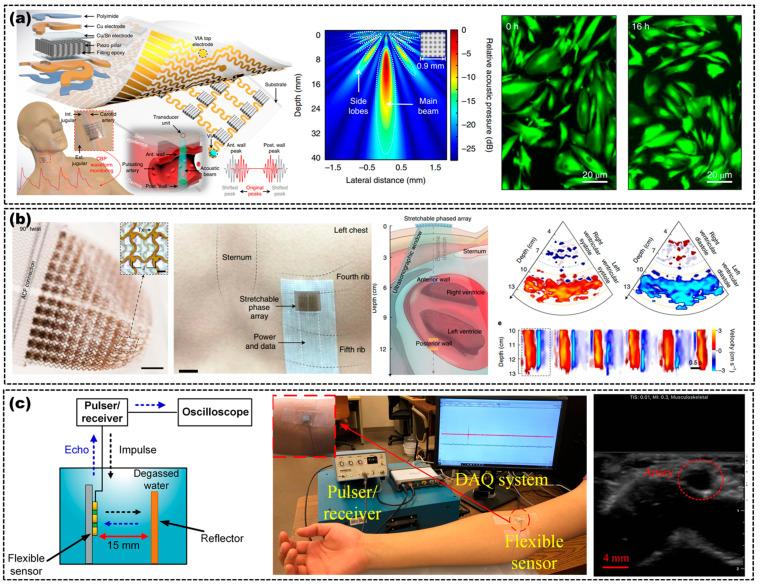
(**a**) Schematic of the transducer on the human chest (**left**), simulated acoustic emission profile of the transducer with excellent beam directivity and penetration depth (**middle**), and fluorescent images of the fibroblast cells before and after 16 h of continuous exposure to the transducer (**right**). Adapted from [56]. (**b**) Optical image of the US transducer (**left**), optical image of the transducer on the human chest (middle), and a schematic and the results of cardiac activity monitoring by the transducer (**right**). Adapted from [22]. (**c**) Schematic of the imaging system based on the flexible US transducer (**left**), an overview of the measurement configuration (**middle**), and the cine loop of US imaging of the ulnar artery (**right**). Adapted from [24].

**Figure 9:**
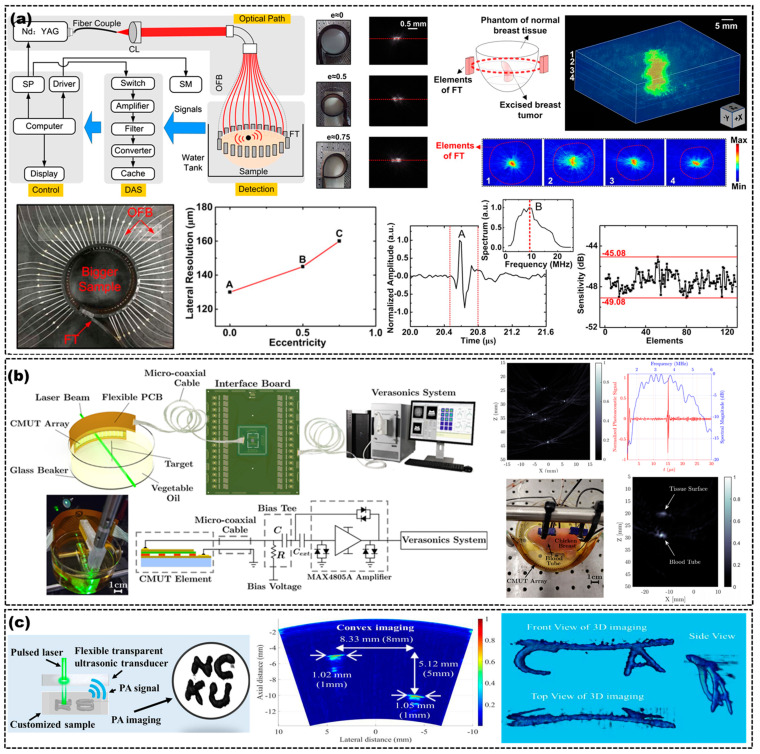
(**a**) Schematic of PAI based on a flexible US array (**top left**), performance characterization of a flexible US transducer (**bottom right**), PA images with different bending US arrays of two truncated hairs (**top middle**), variation between the resolution and the degree of eccentricity of the US array (bottom middle), PA images of breast tumor (**top right**) and optical images of a flexible US array with OFB (**bottom left**). CL, collimating lens; SP, synchronization pulse; SM, stepper motor; OFBs, optical fiber bundles; DAS, data acquisition system. Adapted from [65]. (**b**) Experimental setup for the PAI system (**left**), PA images of wire targets (**top middle**), frequency spectrum of a CMUT in PAI (**top right**), a photo and PA images of chicken breast (middle right). Adapted from [15]. (**c**) Schematic of the PAI system with a flexible US transducer based on PVDF (**left**) and PA images of different objects from different perspectives (**middle** and **right**). Adapted from [26].

US transducers are widely used in imaging applications for their abilities to detect US signals to provide images of target tissues. As the indispensable part of imaging systems, the key parameters of US transducers impact image quality. Imaging systems employing flexible US transduces can be more suitable for the self-alignment to complex surfaces and geometries of the target tissues, compared to that employing rigid US transducers. Flexible US transducers could be used for fixed focus array geometries, tomography arrays, and wrapped around catheter tips for intravascular imaging. Several US imaging and PAI system configurations integrated with flexible US transducers have been reported, as shown in Figure 8 and Figure 9.

Wang et al. designed a flexible US transducer array which can ensure conformal intimate contact with the curvilinear and time-dynamic skin surface, and continuously monitor the central blood pressure of deep vasculatures [56]. The transducer had a center frequency of 7.5 MHz, good sensitivity of 32% bandwidth and excellent beam directivity and 40 mm penetration depth for tissue detection, as shown in Figure 8a. They also used the flexible US transducer to observe the fibroblast cells, which had important implications for biomedical imaging. Wang et al. also fabricated a flexible and stretchable US array for the continuous monitoring of hemodynamic signals from deep tissues up to 14 cm beneath the skin [22]. The SNR along the main beam was demonstrated to be greater than 18 dB in in vitro characterization, as shown in Figure 8b. Peng et al. used a flexible piezocomposite US transducer, with a center frequency of 5 MHz, for continuous blood pressure measurement through US motion tracking of the blood vessel wall, as shown in Figure 8c [24].

Wang et al. used a 128-element flexible US transducer for morphological-adaptive breast detection in a fully flexible PAI system, which had a better resolution and higher energy utilization than a traditional PAI system [65], as shown in Figure 9a. The flexible US transducer was made of BaTiO3, PVDF and a rubber substrate. Figure 9a displays the excellent performance of the flexible US transducer, with a 10 MHz center frequency and 70% bandwidth. The sensitivity of the elements ranged from −45.08 to −49.08 dB. Roy et al. used a flexible 1-D linear US array, based on 750 μm thick PZT-5H, to obtain a PA signal from a PAI system, as shown in Figure 4c. They acquired a 37% fractional bandwidth at −6 dB and a peak voltage of 22 mV at 0 dB [55]. Mahyar et al. bonded a 64-element CMUT array to a flexible PCB to detect PA signals, as shown in Figure 9b. The PAI system with a flexible CMUT array showed an SNR of 46 dB with an axial and lateral resolution of 382 μm and 293 μm, respectively [15]. Liu et al. used a single-element US transducer with a 6.7 MHz center frequency and 86.3% −3 dB fractional bandwidth in a PAI system, as shown in Figure 9c [26]. The PAI system with a flexible US transducer also showed the capability and quality of evaluating the imaging depth and 3D imaging, which could image a 2 mm beam from a distance of 36 mm with a 0.94% error in the axial position and 1.66% in the lateral.

## 5. Discussion and Perspectives

In this review, we presented recent advancements in the development of flexible US transducer and their practical applications in imaging systems. First, we reviewed the functional materials for flexible US transducers, including high performance piezoelectric materials as the active materials, flexible polymer substrates and flexible electrical conductors. Second, we reviewed representative flexible single-element US transducers and common types of flexible array US transducers. Furthermore, we reviewed the configurations and experimental results of the representative research on imaging systems that employed flexible US transducers. Overall, employing flexible US transducers in imaging systems can ensure the self-alignment to the target tissue surfaces, even with complex geometry, so that the imaging systems can detect the maximized transmitted US energy to obtain high-quality images.

As previously discussed, the development of technologies promotes the rise of flexible US transducers. The performance of flexible US transducers systems is mainly affected by several factors, such as bandwidth, sensitivity and center frequency, which also determine the qualities of the images from the imaging systems. Piezoelectric polymers, rigid piezoelectric films and piezocomposites are typical active materials for flexible US transducers. A tradeoff between the different performances of piezoelectric materials should be considered for the requirements of different US transducers.

Piezoelectric polymers usually have a low acoustic impedance and excellent inherent flexibility, which are suitable for fabricating wide bandwidth flexible US transducers without extra matching layers. However, their low electromechanical coupling coefficient and low relative dielectric permittivity would limit their employment in flexible array US transducers. Rigid piezoelectric films, such as PZT and ZnO films, are often deposited on flexible substrates to be used as the active materials for the flexible single-element US transducers due to their simple fabrication processes. Nevertheless, the electromechanical coupling and piezoelectric properties of the rigid piezoelectric films are inferior to bulk rigid piezoelectric materials, which would limit the sensitivity of the flexible US transducers. Moreover, the rigid piezoelectric films usually exhibit high acoustic impedance which is mismatched with the body tissues; thus, US transducers based on rigid films require acoustic matching layers to realize acoustic coupling with body tissues. However, flexible US transducers based on rigid piezoelectric films have been configured without backing layers and matching layers to avoid reducing flexibility and increasing process complexity, which may narrow the bandwidth and affect the quality factor of the transducers. Additionally, rigid piezoelectric films may suffer structural breakage or even fall off from the flexible substrates after multiple deformations, which would also affect the performances of flexible US transducers. Generally, piezocomposites can tailor the performances of the rigid piezoelectric materials and piezoelectric polymers, which are suitable to be used as active materials for flexible US transducers. The flexibility, electromechanical properties and other performances of the piezocomposites are determined by the composition and arrangement of the phases comprising the piezocomposites. However, in fact, the piezocomposites have problems, such as limited flexibility, insufficient polarization, and complicated fabrication processes. Therefore, more research should be undertaken to improve the flexibility, maximize the degree of polarization, and simplify fabrication techniques of the piezocomposites.

Flexible electrical conductors, which are used as the electrodes and electrical connections of the matrix of US transducer units in the flexible US array, are important for the performances of the transducers. There are also some challenges regarding the development of flexible electrical conductors. For instance, the improvement of mechanical flexibility may reduce the charge transfer and eventually lead to the decrease in the conductivity of the flexible electrical conductors. Hence, further investigations are necessary to improve the flexibility while maintaining the electrical conductivity of the flexible electrical conductors. Moreover, conductive polymers have been widely used as flexible electrical conductors for wearable supercapacitors and sensors because of their inherent high flexibility. However, there are few reports on flexible US transducers with conductive polymers as the electrical conductors. In the future, conductive polymers and their composites, with high flexibility and low acoustic impedance, have promising potential in flexible US transducers for imaging applications.

Furthermore, flexible US transducers are mainly based on micromachining manufacture technologies and require complicated fabrication processes, such as the preparation of active materials, the construction of laminated electrical conductors (especially metal-type flexible conductors) and the integration of devices, which will enhance the instability and the risk of the fabrication process to some extent. Further efforts should be made to simplify the fabrication processes to improve the stability and repeatability of the flexible US transducers during processing. Additionally, there is much room for improvement in the center frequency of flexible micromachined US transducers. In the future, further research in optimizing the design of flexible functional materials and transducer structures could be undertaken to maintain their excellent performances after long periods of deformation.

In summary, flexible US transducers are a crucial part of imaging systems and can ensure self-alignment to the complex target tissue surfaces, which is useful for obtaining high-quality images by detecting the maximized transmitted US energy. In the future, with the continuous advancement in the development of flexible functional materials and micromachining manufacture technologies, the challenges of flexible US transducers will be tackled and their applications in the field of imaging will be expanded.

## Data Availability

Not applicable.
